# Therapeutic effects of eye movement desensitization and reprocessing for substance use disorders: a meta-analysis of addiction-related and emotional symptoms

**DOI:** 10.3389/fpsyt.2025.1660046

**Published:** 2025-09-17

**Authors:** Ji-Woo Seok, Kahye Kim, Jaeuk U. Kim

**Affiliations:** ^1^ Digital Health Research Division, Korea Institute of Oriental Medicine, Daejeon, Republic of Korea; ^2^ KM Convergence Science, University of Science and Technology, Daejeon, Republic of Korea

**Keywords:** EMDR, substance use disorder, craving, PTSD, depression, anxiety

## Abstract

**Objective:**

This meta-analysis identified the effects of EMDR on both addiction-related symptoms (e.g., craving, addiction severity) and comorbid emotional symptoms (e.g., posttraumatic stress disorder (PTSD), depression, anxiety), and the influence of moderator variables across these symptom domains in substance use disorders (SUDs).

**Methods:**

We systematically searched the literature published up to June 2025 through major databases including Cochrane, PubMed, Embase, and PsycINFO. A total of 14 studies were included in the final analysis, including randomized controlled trials (RCTs), randomized crossover studies, and quasi-experimental studies. The effect size was calculated using Hedges’ g based on pre-to-post treatment changes, and a meta-analysis was conducted using a random-effects model. In addition, meta-regression and subgroup analyses were performed, focusing on moderator variables such as study design, intervention type, total session number, and participant characteristics.

**Results:**

The meta-analysis results showed that EMDR produced a significant treatment effect with moderate or higher effect sizes for craving (g = 0.55), PTSD (g = 0.69), depression (g = 0.64), and anxiety (g = 0.72) symptoms, and heterogeneity ranged from low to moderate. On the other hand, the effect on addiction severity was not significant (g = 0.14). The effect on craving showed significant differences depending on the diagnostic group of the participants (Alcohol/Drug Use Group vs. Nicotine Use Group) and the study design (RCT vs. non-RCT). Some studies observed a short-term effect of reducing craving, but the evidence supporting long-term treatment effects was limited.

**Conclusion:**

These findings suggest that EMDR may be an effective intervention not only for emotional comorbid symptoms in individuals with SUD, but also for certain addiction-related symptoms, particularly in reducing craving. However, the quality of the included studies was generally low, and there was a lack of evidence regarding long-term effects. Future studies should employ more rigorous research designs, include sufficient sample sizes and long-term follow-up assessments, and perform detailed analyses that take into account intervention types and participant characteristics. Such research will help to clarify the therapeutic utility of EMDR and promote its practical application in addiction treatment settings.

**Systematic review registration:**

https://www.crd.york.ac.uk/PROSPERO/view/CRD420251070837, identifier CRD420251070837.

## 1 Background

Substance use disorder (SUD) occurs when an individual continues to use a substance or alcohol despite the harmful consequences and serious impairments to health, functioning, work, family, or school responsibilities (1). SUDs are emerging as a serious public health problem worldwide. According to statistics from the World Health Organization (WHO), approximately 5.5% of the population aged 15 to 64 worldwide have used psychoactive substances at least once, and approximately 35 million people suffer from drug use disorders (2). SUDs not only affect the quality of life of individuals, but also cause serious social and economic consequences, such as a substantial economic burden on society, increased crime rates, overload of the judicial system, and high mortality rates. In particular, the mortality rate due to synthetic opioid-related overdose has increased recently, altering mortality trends in high-income countries (3).

SUDs affect the brain’s reward circuitry (4). In particular, the mesolimbic dopamine pathway—that is, the circuit connecting the ventral tegmental area (VTA), nucleus accumbens (NAc), and prefrontal cortex (PFC)—plays a central role in the reinforcing effects of drug use and reward responses. This circuitry originally regulates the pleasure and motivation associated with natural rewards such as food, sex, and social stimuli, but repeated drug use induces both functional and structural changes (5). These changes suggest that the mesolimbic dopamine system is not simply a circuit that induces pleasure, but rather a motivational mechanism that assigns incentive salience to certain stimuli or behaviors, making those behaviors “wanted” (6). Alcaro et al. (7) proposed that this circuit functions as a motivational “seeking system”, encouraging the pursuit of survival-promoting behaviors and the avoidance of aversive stimuli. Chronic drug exposure further alters this neural circuit (7). Repeated drug use affects the dopamine and opioid systems, glutamatergic transmission, and cAMP-CREB signaling pathways, resulting in addiction-specific neurobiological mechanisms such as tolerance, dependence, withdrawal, sensitization, and relapse (8). Accordingly, SUDs are considered a chronic condition that is difficult to resolve with short-term interventions and therefore requires early intervention and integrated treatment.

Recent research has emphasized that SUDs are not simply explained by changes in the brain’s reward circuitry, but are closely intertwined with various psychosocial factors, including traumatic experiences, attachment difficulties, and deficits in emotional regulation (9–13). Indeed, a significant number of people with SUD report traumatic experiences, such as physical or sexual abuse in childhood or adulthood, which not only increase the risk of developing PTSD but also disrupt the development of secure attachment (11, 13–15). Empirical studies have shown that childhood physical and emotional abuse or neglect are strongly associated with avoidant attachment style, whereas emotional neglect and sexual abuse are more closely related to anxious attachment style (14, 15). Anxious or avoidant attachment styles are associated with difficulty regulating emotions and heightened impulsivity, which in turn heightens vulnerability to substance use (10, 12). Deficits in emotion regulation abilities hinder the effective management of negative emotions, which can lead to the use of substances as a means of self-medication and the perpetuation of addictive behaviors over time (9, 10).

Comorbid conditions such as PTSD and depression are particularly closely linked to these psychosocial factors and play a central role in the onset and maintenance of SUDs (11, 12). In fact, SUD shows a high comorbidity rate with psychiatric disorders, with PTSD and major depressive disorder (MDD) representing the most common forms (16, 17). Approximately 89% of individuals with SUD who seek treatment have experienced a traumatic event, 11–60% of whom meet criteria for PTSD, while around 85% report mood disorders including MDD (18, 19). The coexistence of PTSD or depression with SUD is associated with significantly poorer prognosis, including higher rates of suicide attempts, chronic physical illness, legal problems, recidivism, and treatment dropout compared with having a single disorder (16, 20, 21).

Although various psychotherapeutic approaches have been attempted for patients with comorbidity, there is still no established standard treatment with consistently demonstrated efficacy (22). Representative treatments currently used include the Seeking Safety program, general cognitive behavioral therapy (CBT), cognitive processing therapy (CPT), prolonged exposure therapy (PE), and trauma-informed CBT including emotion regulation training (21–23). These treatments have shown a certain level of effectiveness in patients with PTSD and SUD, or depression and SUD. However, these treatments are associated with a high treatment dropout rate due to the clinical characteristics of patients with comorbidity, such as emotional sensitivity, low self-control, and avoidant coping styles. Exposure-based therapies, in particular, can lead to emotional exhaustion and symptom exacerbation due to re-experiencing. In addition, it is challenging to apply treatments requiring sustained self-regulation to SUD patients with impaired prefrontal lobe function caused by repeated drug use (21, 22, 24). When PTSD and depression coexist, treatment efficacy may be diminished due to symptom interaction, and limitations have been reported in terms of patient engagement and treatment adherence (22, 23).

Given these limitations, Eye Movement Desensitization and Reprocessing (EMDR) therapy is gaining attention as a promising therapeutic alternative. EMDR is a psychotherapy technique originally developed for the treatment of PTSD. EMDR is a comprehensive psychotherapy approach consisting of an eight-phase protocol (25). This approach is based on the Adaptive Information Processing (AIP) model, which posits that pathological symptoms emerge when traumatic memories remain inadequately processed (26). EMDR’s goal is to facilitate the reprocessing of these memories and their integration into adaptive memory networks. It reduces the emotional intensity of traumatic memories and facilitates cognitive restructuring by inducing memory reprocessing through bilateral stimulation (27). In particular, EMDR has been shown to be effective not only for PTSD but also for various clinical populations, including major depressive disorder, anxiety disorders, somatic symptom and related disorders, and personality disorders (28–31).

Previous case studies and early clinical trials suggest the potential effectiveness of EMDR in treating patients with SUD (24, 32, 33). For example, in a randomized trial using the CravEx approach, only two EMDR sessions led to a large reduction in craving. The EMDR group’s mean OCDS score decreased from 20.4 at baseline to 9.5 post-treatment (T = 10.7, p <.001), whereas the control group showed little change (20.3 to 18.7; T = 1.1, p = .29). At one-month follow-up, EMDR participants still reported significantly lower craving (13.7 vs. 20.9; p <.05), and by six months their relapse rate was lower (67% vs. 100%) (34, 35). In addition, some studies reported significant reductions in the vividness and emotional intensity of substance-related imagery, suggesting the possibility of alleviating craving (36, 37). However, these findings are mostly based on small-scale studies or single-case designs, and there are limitations due to the lack of long-term follow-up data and rigorous control group comparisons.

Recently, some systematic reviews and meta-analyses have been conducted on the application of EMDR to addiction populations (3, 21, 70). According to a narrative review, the effects of EMDR on PTSD symptoms were consistent, but its effects on SUD-related outcomes (e.g., craving, relapse, symptom severity) were often inconsistent or inconclusive (21). Logsdon et al. (70) conducted the first SUD-specific EMDR meta-analysis and reported an overall effect size (Hedges’ g = 0.654, p <.001), with particularly large effects observed for comorbid symptoms such as PTSD (g = 1.426) and depression (g = 0.93). However, the number of included studies on SUD-specific indicators (e.g., craving, relapse rate, treatment participation) was small, which contributed to substantial heterogeneity in effect sizes (70). A recent systematic review and meta-analysis found that EMDR treatment significantly reduced craving in SUD patients; the standardized mean difference (SMD) based on the fixed-effects model was reported as −0.866 (95% CI = −1.121 to −0.611, z = −6.66, p <.0001). Although this finding suggests that EMDR may be an effective intervention for reducing craving, the study has limitations in that it was not a meta-analysis that included other symptom domains or conducted a comprehensive symptom-level analysis (3).

In addition, most existing meta-analyses have limitations in that they calculate effect sizes solely based on the post-treatment mean differences between experimental and control groups (3, 70). This analytic approach does not provide a precise comparison of within-group pre- to post-treatment changes, and it also has limitations in interpreting the clinical significance of treatment effects. Therefore, this study independently analyzed the pre-post change within each group, and then compared the differences in these changes between groups through meta-analysis to provide a more rigorous assessment of treatment efficacy.

Therefore, the purpose of this meta-analysis is to comprehensively evaluate the effects of EMDR on the following major domains in patients with SUD: (1) SUD-specific symptoms (e.g., craving, addiction severity), and (2) comorbid psychiatric symptoms (e.g., PTSD, depression, anxiety). In addition, moderator variables such as the type of target memory in EMDR (trauma vs. addiction), number of sessions, clinical population, and type of control group are also analyzed to provide practical guidance for future clinical practice.

## 2 Methods

### 2.1 Study design

This research is a meta-analysis and meta-regression study aimed at assessing the efficacy of EMDR on addiction severity, craving, PTSD related symptoms, and emotional problems (depression and anxiety).

### 2.2 Selection and exclusion criteria

This study followed Preferred Reporting Items for Systematic Reviews and Meta-Analyses (PRISMA) guidelines and was registered in International Prospective Register of Systematic Reviews (PROSPERO) (registration number: CRD420251070837). The selection criteria were established based on the Population, Intervention, Comparator, Outcomes (PICO) framework.

The study population included individuals of all ages diagnosed with SUD, regardless of co-occurring mental health conditions such as PTSD, depression, or anxiety, provided that SUD was the primary diagnosis or the main focus of the intervention. The intervention included studies in which EMDR therapy was applied in a clearly identifiable and standardized format. EMDR was considered both as a standalone treatment and when combined with structured psychotherapies such as CBT, Dialectical Behavior Therapy, Seeking Safety intervention, or schema therapy, as long as the control group received the same structured psychotherapy and the study design allowed for the isolation of EMDR’s specific effects. Eligible comparators consisted of non-EMDR conditions, including treatment-as-usual (TAU), waitlist control, supportive counseling, and psychoeducation. Single-group studies were included only if a clearly defined control group was available for comparison; pre-post designs without a comparison group were excluded. The primary outcomes included SUD-related indicators such as addiction severity, craving level, relapse rate, and treatment adherence, in co-occurring emotional conditions such as PTSD, depression, and anxiety. Eligible study designs included randomized controlled trials (RCTs), randomized crossover trials, and quasi-experimental studies.

The exclusion criteria were as follows: studies in which EMDR was not the primary intervention; studies with designs that did not allow for independent estimation of EMDR’s effects; studies employing non-standardized or modified EMDR-based techniques; studies targeting behavioral addictions unrelated to substance use (e.g., gambling, internet addiction); single-group studies without a comparator; case reports; protocol papers; exploratory studies lacking outcome data; and articles not published in English.

### 2.3 Data search and selection process

A systematic search of the literature was conducted based on a predefined strategy. The main search databases included the Cochrane Central Register of Controlled Trials (CENTRAL), Embase, MEDLINE, PsycINFO, PubMed, Science Citation Index (SCI), and Social Science Citation Index (SSCI). An additional search was also performed in ProQuest Dissertations & Theses Global (PQDT Global) to include doctoral and master’s theses.

The search terms were composed mainly of terms related to EMDR, SUD, addiction, and craving, and the main combinations are as follows: (“addiction” OR “substance use disorder” OR “alcohol use disorder” OR “SUD” OR “AUD” OR “craving”) AND (“EMDR” OR “Eye Movement Desensitization and Reprocessing” OR “eye movement psychotherapy”) AND (“Control” OR “waitlist” OR “TAU” OR “treatment as usual” OR “no intervention” OR “CAU” OR “care as usual”). The search was restricted to English-language publications only, and no limitations were placed on the publication year. In addition to the published literature, supplementary records were identified through reference list checking of included articles and backward and forward citation tracking. When necessary, study authors were contacted directly to request missing data.

The study selection process was conducted by two or more reviewers who independently screened titles and abstracts, followed by full-text review to determine eligibility. Discrepancies in study inclusion were resolved through discussion with a third reviewer. The overall screening process was visually presented using the PRISMA 2020 flow diagram.

### 2.4 Quality assessment of included studies

Different risk of bias assessment tools were used according to the study design to assess the methodological quality of the included studies. For randomized controlled trials, we applied the Cochrane Risk of Bias 2.0 (RoB 2.0) tool, and for crossover design studies, we used the RoB 2.0 version for crossover trials. For non-randomized studies, we used the Risk of Bias in Non-randomized Studies of Interventions (ROBINS-I) tool (38, 39).

RoB 2.0 assessed the following five domains of bias: (1) the randomization process, (2) deviations from intended interventions, (3) missing outcome data, (4) bias in the measurement of outcomes, and (5) bias in the selection of the reported result. Each domain was rated as ‘low risk’, ‘some concerns’, or ‘high risk’, and the overall risk of bias was determined based on these domain-level judgments.

For crossover studies, the RoB 2.0 tool specific to crossover trials was used to assess the following six domains: (1) bias arising from the randomization process, (2) bias due to period and carryover effects, (3) deviations from intended interventions, (4) missing outcome data, (5) bias in the measurement of outcomes, and (6) bias in the selection of the reported result. Each domain was likewise rated as ‘low risk’, ‘some concerns’, or ‘high risk’.

For non-randomized studies, the ROBINS-I tool was applied to assess the following seven domains: (1) confounding at baseline, (2) bias in the classification of interventions, (3) deviations from intended interventions, (4) missing outcome data, (5) bias in the measurement of outcomes, (6) bias in the selection of the reported result, and (7) overall risk of bias. Each domain was rated as ‘low’, ‘moderate’, ‘serious’, or ‘critical’.

Quality assessments were performed independently by at least two reviewers. In cases of disagreement, consensus was reached through discussion, or discrepancies were resolved by a third reviewer.

### 2.5 Data extraction

A coding framework for data extraction was developed based on relevant literature, and two research assistants systematically extracted relevant information according to the framework. Extracted items included the first author and year of publication of the study, subject characteristics (e.g., type of substance use disorder and presence of comorbid disorders), study design type (randomized controlled trial, crossover trial, or quasi-experimental study), mean age, composition of intervention and control groups, intervention content (form of EMDR application and whether a concurrent intervention was included), specific intervention protocols such as weekly frequency and duration per session, total number of intervention sessions, and main assessment tools used. Outcome variables included various psychometric measures reflecting the effects of the intervention, such as addiction severity, addiction-related craving, depression, anxiety, PTSD symptoms, emotional and cognitive responses, and overall functioning. The specific tools and subscales used in each study are summarized in **Table 1**.

**Table 1:** Study characteristics of 14 studies selected for the meta-analysis.

Author (year)	Subjects	Reported phase	Study design	Age	Groups	Intervention protocol	Measurement timepoints	Psychometric instruments reported
Carletto et al.(40)	SUD	NR	quasi-experimental study	T: 32 ± 8 C: 32 ± 19	T: Combined trauma- and addiction-focused EMDR+TAU (n=20), C: TAU (n=20)	1 sessions/week, 50 minutes/session, 24 weeks (Total session number, 24)	Baseline, Post-treatment	Craving: NR; Depression: BDI-II; Trauma: DES & IES-R; Addiction Severity: NR; Anxiety: STAI-1 & STAI-2; Emotion Regulation: NR; Other: SCL-90
Hase et al. (35)	AUD	Withdrawal/Detox phase	RCT	T: 45.7 ± 5.2 C: 42.5 ± 8.5	T: AF-EMDR+TAU (n=15), C: TAU (n=15)	NR sessions/week, NR minutes/session, NR weeks (Total session number, 2)	Baseline, Post-treatment, 1-month FU, 6-month FU	Craving: OCDS; Depression: BDI; Trauma: NR; Addiction Severity: NR; Anxiety: NR; Emotion Regulation: NR; Other: NR
Kutsukos (41)	SUD + Trauma	NR	RCT	NR	T: TF-EMDR+TAU (n=12), C: TAU (n=12)	1 sessions/week, 60 minutes/session, under 10 weeks (Total session number, 8)	Baseline, Post-treatment	Craving: NR; Depression: BDI-II; Trauma: PCL-5; Addiction Severity: NR; Anxiety: BAI; Emotion Regulation: NR; Other: CESI
Lemkes et al. (42)	AUD	Post-detox/Early abstinence (inpatient)	RCT	T: 47.9 ± 14.7 C: 47.7 ± 13.2	T: AF-EMDR (n=29), C: Sham (n=21)	NR sessions/week, NR minutes/session, NR weeks (Total session number, 1)	Baseline, Post-treatment	Craving: VAS-Emo, VAS-Cr & VAS-Viv; Depression: NR; Trauma: NR; Addiction Severity: NR; Anxiety: NR; Emotion Regulation: NR; Other: NR
Littel et al. (36)	Daily smokers	NR	quasi-experimental	T: 23.4 ± 6.6 C: 23.4 ± 6.6	T: AF-EMDR + Recall (n=22), C: Recall Only (n=28)	NR sessions/week, NR minutes/session, NR weeks (Total session number, 1)	Baseline, Post-treatment	Craving: VAS-Emo, VAS-Cr, VAS-Viv & QSU-Brief; Depression: NR; Trauma: NR; Addiction Severity: NR; Anxiety: NR; Emotion Regulation: NR; Other: NR
Lortye et al. (63)	SUD + PTSD	NR	RCT	T: 36.34 ± 11.10 C: 38.24 ± 11.75	T: TF-EMDR+TAU (n=50), C: TAU (n=51)	2 sessions/week, 90 minutes/session, 6 weeks (Total session number, 12)	Baseline, 3-month FU	Craving: NR; Depression: NR; Trauma: CAPS-5; Addiction Severity: AUDIT & DUDIT; Anxiety: NR; Emotion Regulation: NR; Other: NR
Markus et al. (37)	Daily smokers	NR	RCT	T: 34.54 ± 14.73 C: 29.61 ± 11.62	T: AF-EMDR (n=24), C: Sham (n=23)	NR sessions/week, NR minutes/session, NR weeks (Total session number, 1)	Baseline, Post-treatment, 1-week FU	Craving: Likert-Craving, VAS-Viv & QSU-Brief; Depression: NR; Trauma: NR; Addiction Severity: NR; Anxiety: NR; Emotion Regulation: NR; Other: NR
Markus et al. (43)	AUD	Relapse prevention/Maintenance	RCT	T: 47.9 ± 11.4 C: 46.3 ± 12.0	T: AF-EMDR + TAU (n=55), C: TAU (n=54)	1 sessions/week, 90 minutes/session, 7 weeks (Total session number, 7)	Baseline, Post-treatment, 1-month FU, 6-month FU	Craving: PACS; Depression: NR; Trauma: NR; Addiction Severity: AUDIT; Anxiety: NR; Emotion Regulation: NR; Other: CRA-HS & EQ-5D
Meysami-Bonab et al. (2012)	SUD + Trauma	Post-detox Rehabilitation	RCT	T: 30.2 C: 29.93	T: TF-EMDR (n=15), C: no treatment (n=15)	NR sessions/week, NR minutes/session, NR weeks (Total session number, 8)	Baseline, Post-treatment	Craving: NR; Depression: NR; Trauma: NR; Addiction Severity: NR; Anxiety: NR; Emotion Regulation: CERQ-Pos & CERQ-Neg; Other: EmotionRecog
Perez-Dandieu and Tapia (19)	SUD + PTSD	NR	RCT	T: 29.67 ± 3.14 C: 29.33± 2.94	T: TF-EMDR (n=6), C: TAU (n=6)	NR sessions/week, NR minutes/session, 24 weeks (Total session number, 8)	Baseline, Post-treatment	Craving: NR; Depression: BDI-II; Trauma: PCL-S; Addiction Severity: ASI; Anxiety: STAI-1; Emotion Regulation: NR; Other: NR
Rooijmans et al. (44)	Heavy smoker	NR	randomized cross-over trial	T: 22.25 ± 3.31 C: 22.25 ± 3.31	T: AF-EMDR (n=36), C: Sham (n=36)	1 sessions/week, 3 minutes/session, 1 week (Total session number, 1)	Baseline, Post-treatment	Craving: VAS-Ple, VAS-Cr & VAS-Viv; Depression: NR; Trauma: NR; Addiction Severity: NR; Anxiety: NR; Emotion Regulation: NR; Other: NR
Sargin et al. (59)	AUD	NR	RCT	T: 41.75 ± 9.15 C: 45.66 ± 11.08	T: AF-EMDR+TAU (n=12), C: TAU (n=12)	1 sessions/week, 60–90 minutes/session, 3 weeks (Total session number, 3)	Baseline, Post-treatment, 6-month FU	Craving: CEQ-Imag & CEQ-Int, PACS; Depression: NR; Trauma: NR; Addiction Severity: NR; Anxiety: NR; Emotion Regulation: NR; Other: GAF
Sgualdini et al. (60)	SUD	Residential Detox/Rehabilitation (early phase)	RCT	NR	T: AF-EMDR+TAU (n=16), C: TAU (n=14)	1 sessions/week, NR minutes/session, 4 weeks (Total session number,4)	Baseline, Post-treatment, 1-month FU	Craving: VAS-Ple, VAS-Unp & VAS-Cr; Depression: NR; Trauma: NR; Addiction Severity: NR; Anxiety: NR; Emotion Regulation: NR; Other: NR
Woodruff et al. (45)	SUD	NR	RCT	T: 40.07 ± 13.32 C: 40.07 ± 13.32	T: AF-EMDR + CBT (n=15), C: CBT Only (n=15)	2 sessions/week, 60 minutes/session, 4 weeks (Total session number, 4)	Baseline, Mid-treatment, Post-treatment	Craving: BSCS; Depression: NR; Trauma: NR; Addiction Severity: NR; Anxiety: NR; Emotion Regulation: NR; Other: MATS & PTQ

AF, Addiction Focused; ASI, Addiction Severity Index – Lite Version; AUD, Alcohol Use Disorder; AUDIT, Alcohol Use Disorders Identification Test; BAI, Beck Anxiety Inventory; BDI, Beck Depression Inventory; BDI-II, Beck Depression Inventory – Second Edition; BSCS, Brief Substance Craving Scale; CAPS-5, Clinician-Administered PTSD Scale for DSM-5; CEQ-Imag, Craving Experience Questionnaire – Imagery; CEQ-Int, Craving Experience Questionnaire – Intensity; CERQ-Neg, Cognitive Emotion Regulation Questionnaire – Negative; CERQ-Pos, Cognitive Emotion Regulation Questionnaire – Positive; CESI, Coopersmith Self Inventory; CRA-HS, Community Reinforcement Approach – Happiness Scale; C, Control group; DES, Dissociative Experiences Scale; DUDIT, Drug Use Disorders Identification Test; EMDR, Eye Movement Desensitization and Reprocessing; EQ-5D, EuroQol-5 Dimensions; EmotionRecog, Emotion Recognition Test; GAF, Global Assessment of Functioning; IES-R, Impact of Event Scale – Revised; Likert-Craving, Likert Scale – Cue-induced Craving; MATS, McMullin Addiction Thought Scale; OCDS, Obsessive–Compulsive Drinking Scale; PACS, Penn Alcohol Craving Scale; PCL-5, PTSD Checklist for DSM-5; PCL-S, PTSD Checklist – Civilian Version; PTSD, Post-Traumatic Stress Disorder; PTQ, Perseverative Thinking Questionnaire; QSU-Brief, Brief Questionnaire on Smoking Urges; SCL-90 GSI, Symptom Checklist-90-Revised (Global Severity Index); SUD, Substance Use Disorder; STAI-1, State-Trait Anxiety Inventory – Form Y1 (State Anxiety); STAI-2, State-Trait Anxiety Inventory – Form Y2 (Trait Anxiety); TAU, Treatment As Usual; TF-EMDR, Trauma Focused EMDR; T, Treatment group; VAS-Cr, Visual Analog Scale – Craving; VAS-Emo, Visual Analog Scale – Emotionality; VAS-Ple, Visual Analog Scale – Pleasantness; VAS-Unp, Visual Analog Scale – Unpleasantness; VAS-Viv, Visual Analog Scale – Vividness.

Data extraction was performed independently by two researchers, and in cases of discrepancies, consensus was reached through discussion or resolved with input from a third reviewer. The extracted data were used in meta-analytic procedures including effect size estimation, subgroup analyses, and meta-regression analyses.

### 2.6 Statistical analysis

In this study, effect sizes (standardized mean differences, Hedges’ g) were extracted from each selected study, and a meta-analysis was performed based on a random-effects model (46). The analysis was conducted separately for each major outcome variable, including depression, anxiety, craving, PTSD symptoms, and addiction severity. The effect size was calculated based on the difference in change scores between the intervention and control groups, and the standardized mean difference (SMD) was computed according to the recommendations in the Cochrane Handbook (47). When the standard deviation of the change score was not available, the effect size was conservatively estimated by assuming a pre-post correlation of zero. Hedges’ g was then calculated to correct for small sample bias, and the standard error was also estimated (47).

All statistical analyses were performed using JASP software (v0.19.0.0), and effect sizes were estimated using the restricted maximum likelihood (REML) method (48, 49). Between-study heterogeneity was assessed using Cochran’s Q statistic, I², τ², and H² indices (50, 51).

Publication bias was assessed both visually using funnel plots and statistically using Egger’s regression test, Kendall’s τ, and Orwin’s Fail-safe N. Additionally, influence analyses using standardized residuals, DFFITS, and Cook’s Distance were performed to evaluate the impact of individual studies on the overall results (52–56). A meta-regression analysis was conducted to explore potential sources of heterogeneity, with the total number of intervention sessions included as a continuous moderator variable (57). The significance of the regression model and its contribution to reducing heterogeneity were examined using Wald tests and omnibus tests of model coefficients (58).

Furthermore, subgroup analyses were conducted to examine potential differences in treatment effects. Subgroups were defined according to participant characteristics (e.g., presence of comorbid mental disorders), type of EMDR application (e.g., addiction-focused, AF vs. trauma-focused, TF), type of control group (e.g., waitlist, treatment-as-usual), and study design (e.g., RCT, crossover, quasi-experimental design). All subgroup analyses were conducted using a random-effects model with the REML method, and results were reported with corresponding effect sizes and heterogeneity statistics.

## 3 Results

### 3.1 Selection of studies

**Figure 1:**
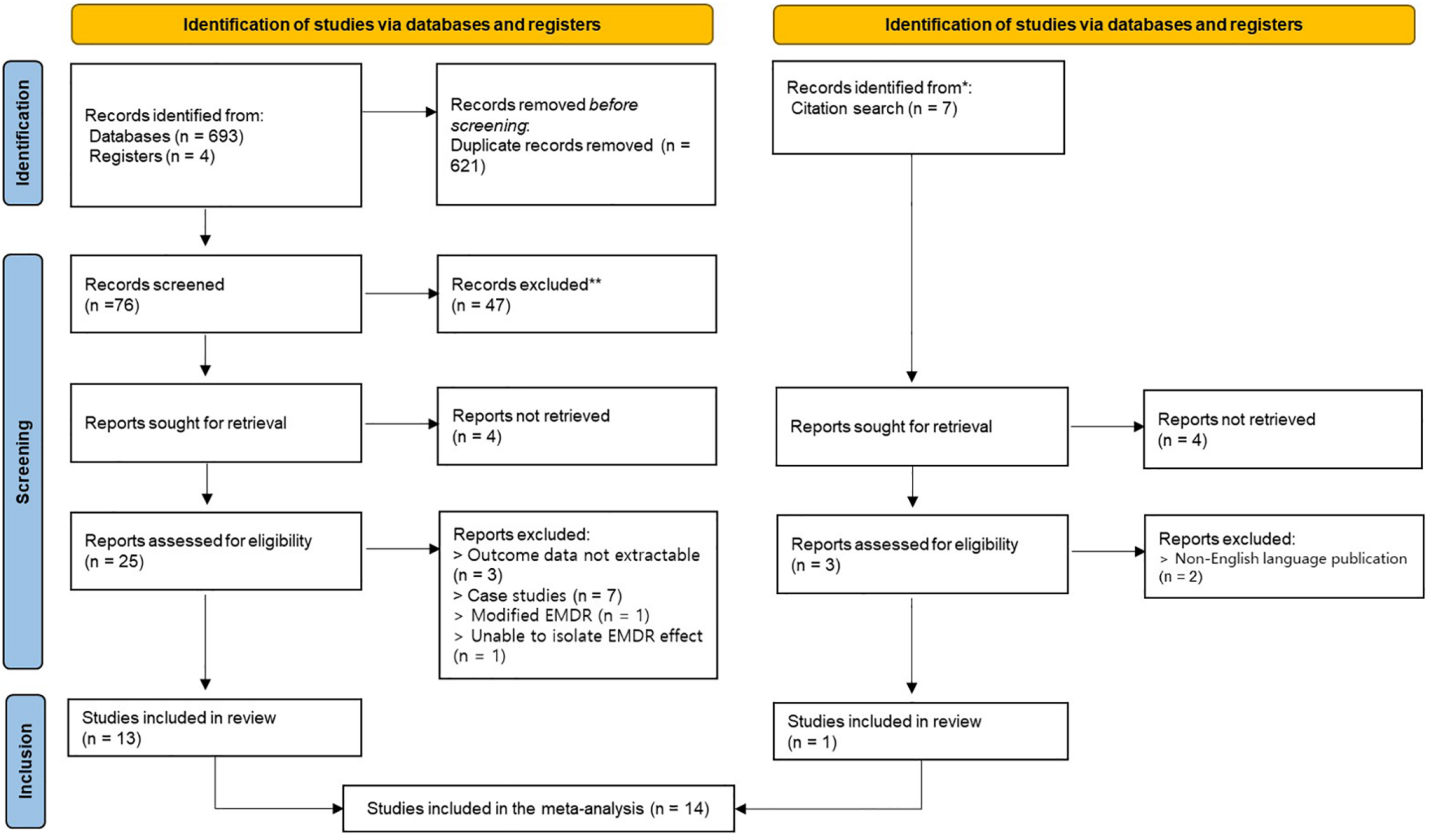
Flow diagram of study selection.

A total of 704 articles were identified through the literature search, of which 697 were identified through database and registry searches and 7 through citation searches. In the initial stage, 621 duplicate articles were removed, and the remaining 76 articles were screened based on the inclusion and exclusion criteria at the title and abstract level. As a result, 47 articles were excluded, and a total of 28 articles were selected for full-text review. Of these, 12 articles were excluded for the following reasons: 3 articles did not provide sufficient statistical information to calculate effect sizes, 7 articles were classified as case studies, 1 article used a modified form of EMDR, and 1 article did not allow isolation of the EMDR effect. Of the 7 articles identified through the citation search, 3 were fully evaluated, and 2 of them were excluded because they were not published in English. Finally, 14 studies were included in this systematic review, yielding a total of 22 effect sizes that were synthesized in the meta-analysis (see **Figure 1**).

### 3.2 Characteristics of the studies

The main characteristics of the 14 studies included in this meta-analysis are summarized in **Table 1** (9, 19, 35–37, 40–45, 59, 60). Each study included information on intervention type, participant characteristics, mean age, sample size, type of control group, intervention frequency and duration, total number of sessions, and main assessment tools.

The included studies were published between 2008 and 2025, and all targeted alcohol/substance use disorders or nicotine dependence. Among them, 4 out of 14 studies included participants with trauma exposure or PTSD. All studies involved adults, and no studies involved children or adolescents. The total sample size was similar, with 327 participants in the EMDR intervention group and 322 participants in the control group. The types of interventions were broadly classified into three types based on the focus of application. First, Addiction-focused EMDR (AF-EMDR) was used in a total of nine studies, and was an intervention method that mainly focused on directly addressing alcohol or nicotine addiction. Second, Trauma-Focused EMDR (TF-EMDR) was used in four studies and consisted of an intervention centered on traumatic experiences or PTSD history. Finally, one study applied a combined EMDR intervention that reflected both addiction and trauma elements (40).

The control group varied from treatment as usual (TAU), sham conditions, a group that received only reminiscence, to a group that received only CBT. The number of sessions ranged from 1 to 12, and some studies reported the average number of sessions. The typical duration per session was 50 to 90 minutes, and the frequency was 1–2 times per week. Each study evaluated the intervention effects through various psychological and behavioral indicators. Scales used to assess the severity of addiction included the Alcohol Use Disorders Identification Test (AUDIT), Addiction Severity Index (ASI), Drug Use Disorders Identification Test (DUDIT), and Obsessive Compulsive Drinking Scale (OCDS). Craving-related assessments included the Visual Analog Scale for Craving (VAS-Cr), the Questionnaire on Smoking Urges (QSU-Brief), Likert-type craving ratings, and the Penn Alcohol Craving Scale (PACS). Depressive symptoms were primarily measured using the Beck Depression Inventory-II (BDI-II). Scales related to PTSD included the Posttraumatic Stress Disorder Checklist (PCL-5, PCL-S), the Impact of Event Scale-Revised (IES-R), and the Clinician-Administered PTSD Scale for DSM-5 (CAPS-5). Additionally, the State-Trait Anxiety Inventory (STAI-1, STAI-2) and Beck Anxiety Inventory (BAI) were used to assess anxiety levels.

### 3.3 Quality assessment results

The risk of bias of the included studies was assessed using the ROB 2.0 (Risk of Bias 2) and ROBINS-I tools according to the study design, and the results are summarized in **Supplementary Figure S1**.

A total of 11 randomized trials were assessed using ROB 2.0. Of these, 7 were classified as ‘high risk’ overall, 2 as ‘some concerns’, and 2 as ‘low risk of bias’. In particular, the domains where the risk of bias was most prominent were D3 (handling of missing outcome data), D5 (selection of reported results), and D1 (randomization process). In D3, a total of 7 studies were identified as having a risk of bias based on the responses of ‘Yes’ or ‘Probably yes’, and in D5, problems were identified in 3 studies. On the other hand, the D4 (appropriateness of outcome measurement tools) domain was evaluated as ‘low risk’ in all 11 studies, indicating relatively robust outcome assessment across studies.

One randomized crossover trial was assessed using the ROB 2.0 tool adapted for crossover designs (44). This study was judged as ‘high risk’ overall due to concerns in the randomization process (D1) and period and carryover effects (D5), despite showing low risk in most other domains. In addition, two non-randomized design studies were assessed using ROBINS-I, and both studies were evaluated as having an overall serious risk of bias (36, 40). The main sources of bias were confounding, participant selection, and bias in outcome measurement. These quality assessment results should be interpreted with caution and are visually presented in **Supplementary Figure S1**.

### 3.4 The effect of EMDR on craving

**Figure 2:**
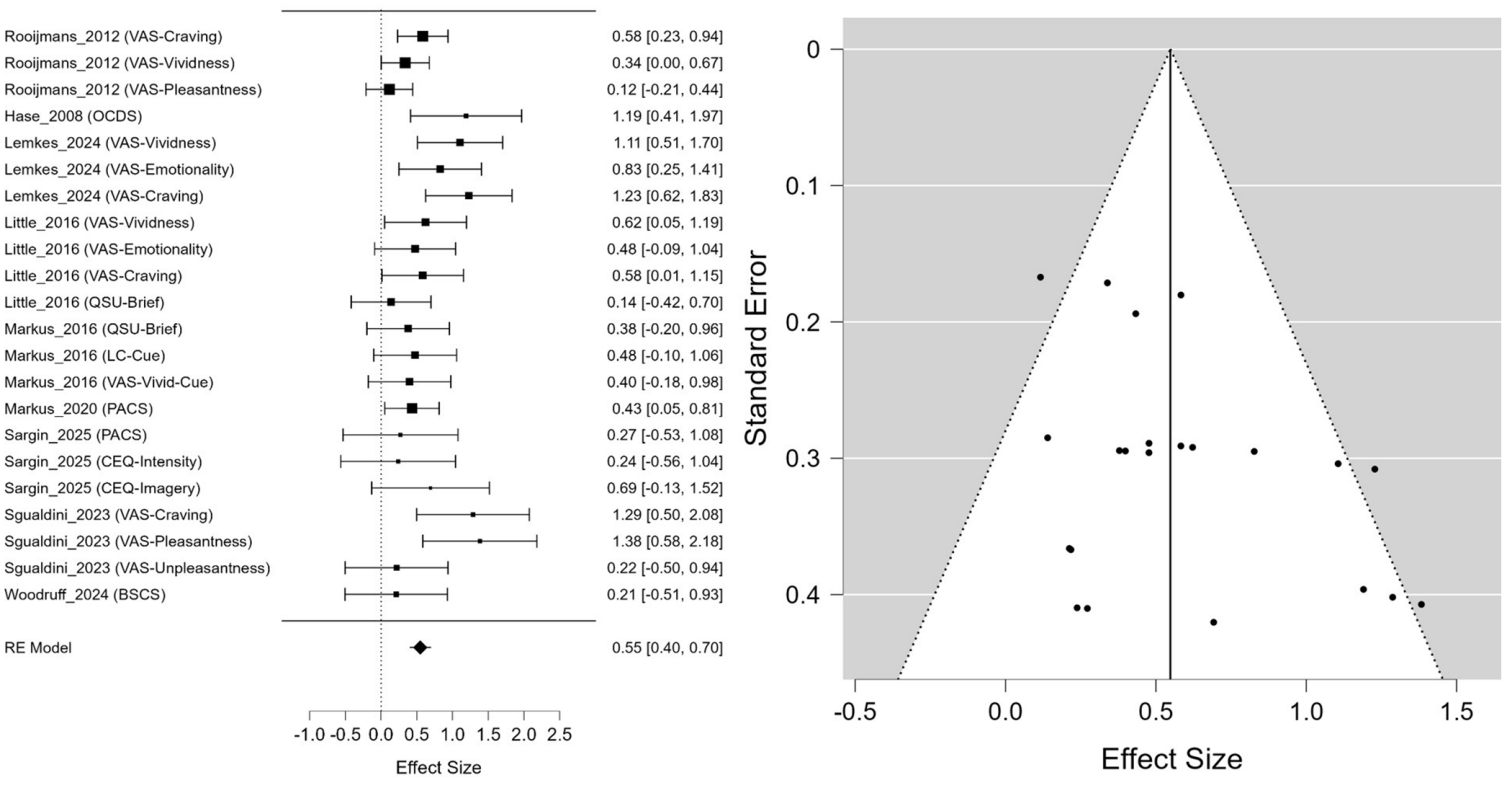
Forest plot and funnel plot for craving outcomes.

The meta-analysis, which included a total of 22 effect sizes derived from 14 studies, showed that EMDR intervention had a statistically significant positive effect on reducing craving. The estimated mean effect size was Hedges’ g = 0.548 (95% CI: 0.399, 0.697, p <.001) (**Figure 2**). The heterogeneity analysis indicated a moderate degree of heterogeneity, with I² = 34.95%, which was also statistically supported by the result of the residual heterogeneity test (Q(21) = 33.113, p = .045).

As for publication bias, Egger’s regression test revealed statistically significant asymmetry (z = 2.264, p = .024), suggesting the possibility of some publication bias. In contrast, Kendall’s rank correlation test was not statistically significant (τ = 0.299, p = .054), making it difficult to conclude that publication bias was clearly present. In addition, Rosenthal’s fail-safe N was 654, indicating that the observed results were unlikely due to chance and were relatively robust against potential publication bias.

The results of the meta-regression analysis exploring moderators of the effect size for craving were as follows. Participant characteristics were identified as statistically significant moderator variables (Omnibus Q(2) = 8.571, p = .014), and the effect size in the AUD group was significantly larger than in the smoker group (β = 0.387, 95% CI [0.117, 0.657], p = .005). In contrast, the other SUD group did not show a significant difference from the smoker group (β = −0.178, 95% CI [−0.955, 0.600], p = .655).

The type of study design also emerged as a significant moderator (Omnibus Q(1) = 3.982, p = .046), with studies using crossover or quasi-experimental designs showing a significantly smaller effect size compared to RCTs (β = −0.278, 95% CI [−0.551, −0.005], p = .046). In contrast, neither the type of control group (active vs. passive) nor the total number of intervention sessions showed a statistically significant moderating effect (p >.05) (**Table 2**).

**Table 2:** Results of meta-regression analyses for moderator variables of EMDR effects on craving.

Moderator variable	Level	Reference level	β	95% CI	*z*	*p*-value	Omnibus Q(df)	Omnibus *p*
Participant type	AUD	Smoker	0.387	[0.117, 0.657]	2.806	0.005^**^	8.571 (2)	0.014^*^
	Other SUD		-0.178	[−0.955, 0.600]	-0.447	0.655		
Control type	Active	Passive	0.008	[−0.299, 0.315]	0.052	0.958	0.003 (1)	0.958
Study design	Randomized crossover OR Quasi experimental study	Randomized controlled trials	-0.278	[−0.551, -0.005]	-1.995	0.046^*^	3.982 (1)	0.046^*^
Total session number	–	Continuous	0.003	[-0.085, 0.092]	0.075	0.940	0.006 (1)	0.940

CI, confidence interval; AUD, alcohol use disorder; SUD, substance use disorder; RCT, randomized controlled trial.

For each categorical moderator, the reference level is indicated in the second column. The regression coefficient (β) represents the estimated difference in effect size compared to the reference group.

Omnibus Q and *p*-values refer to the overall significance of each moderator variable. The total session number (frequency per week × duration in weeks) was entered as a continuous predictor.

*p* <.05 was considered statistically significant and is marked with an asterisk (*); *p* <.01 is marked with two asterisks (**).

### 3.5 The effect of EMDR on addiction severity

**Figure 3:**
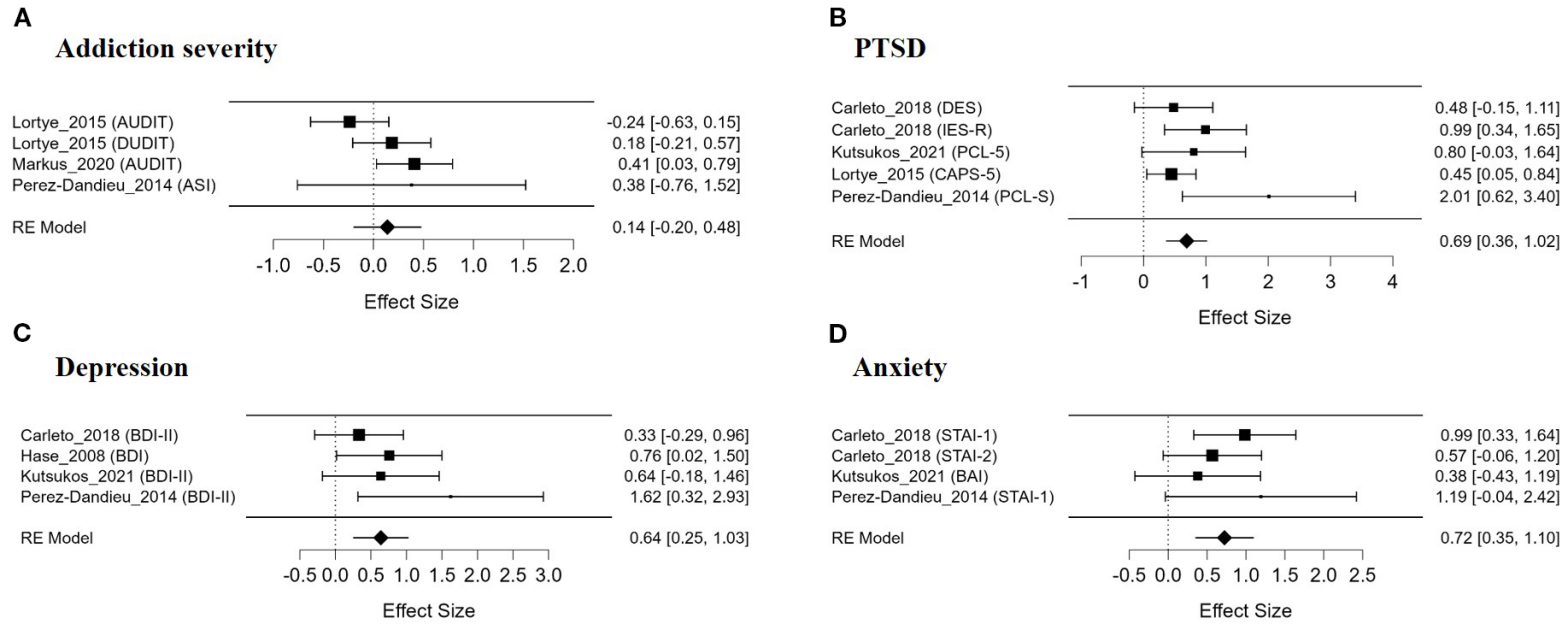
Forest plots for each symptom domain.

The meta-analysis evaluating the effect of EMDR on addiction severity did not yield a statistically significant overall effect size (**Figure 3A**). The average effect size was Hedges’ g = 0.140, which was not statistically significant (SE = 0.172, z = 0.815, p = .415). The overall test of model coefficients (Omnibus test) also did not indicate statistical significance (Q(1) = 0.664, p = .415). The residual heterogeneity test did not reveal statistically significant heterogeneity, but a moderate level of heterogeneity was observed (Q(3) = 5.715, p = .126). The heterogeneity index I² was 50.95%, indicating moderate heterogeneity, and τ² was estimated at 0.057.

In the publication bias analysis, Egger’s regression test showed no statistically significant evidence of funnel plot asymmetry (z = 0.363, p = .717), and Kendall’s rank correlation coefficient likewise indicated no significant asymmetry (τ = −0.333, p = .750), suggesting a low likelihood of publication bias. Overall, EMDR did not demonstrate a significant effect in reducing addiction severity. Despite the moderate between-study heterogeneity, the potential influence of publication bias or outliers appears to be minimal. It should be noted that the included studies did not consistently differentiate between specific SUD subtypes (e.g., alcohol, opioids, nicotine), and thus the results were synthesized across SUDs as a whole.

### 3.6 The effect of EMDR on PTSD symptom

The meta-analysis assessing the efficacy of EMDR on PTSD symptoms in individuals with addiction revealed a moderate and statistically significant overall effect size (**Figure 3B**). The mean effect size was Hedges’ g = 0.692, which was statistically significant (SE = 0.167, z = 4.146, p <.001). The omnibus test of model coefficients also yielded statistically significant results (Q(1) = 17.189, p <.001). Despite the absence of statistically significant heterogeneity in the residual heterogeneity test (Q(4) = 6.167, p = .187), a small degree of between-study heterogeneity was observed, as indicated by a heterogeneity index of I² = 19.86% and an estimated τ² = 0.029.

In the investigation of publication bias, Egger’s regression test revealed statistically significant funnel plot asymmetry (z = 2.178, p = .029), indicating a potential risk of publication bias. However, Kendall’s rank correlation test was not statistically significant (τ = 0.800, p = .083). Furthermore, Rosenthal’s fail-safe N was 44, suggesting that the likelihood of the observed results being attributable to chance is low. In conclusion, EMDR demonstrated a moderate and reliable effect in alleviating PTSD symptoms in patients with substance use disorders. The potential influence of publication bias or outliers was assessed to be relatively limited.

### 3.7 The effect of EMDR on depressive symptom

The meta-analysis evaluating the treatment effect of EMDR on depressive symptoms in patients with substance addiction identified a statistically significant overall effect size (**Figure 3C**). The average effect size was Hedges’ g = 0.640, which was statistically significant (SE = 0.201, z = 3.190, p = .001, 95% CI [0.247, 1.034]). The overall test of model coefficients (Omnibus test) also yielded statistically significant results (Q(1) = 10.177, p = .001). In the residual heterogeneity test, no statistically significant between-study heterogeneity was observed (Q(3) = 3.204, p = .361), and the heterogeneity index I² was extremely low (0.00069%), indicating negligible heterogeneity. τ² was estimated to be very close to zero (0.0000012).

In the publication bias analysis, Egger’s regression test did not detect statistically significant asymmetry, but a marginal p-value was reported (z = 1.713, p = .087). Kendall’s rank correlation test was also not statistically significant (τ = 0.667, p = .333). Rosenthal’s fail-safe N was 15, suggesting that the results are unlikely to be attributable to chance and exhibit a moderate level of robustness. In summary, EMDR was found to have a moderate and statistically significant effect on reducing depressive symptoms in SUD patients, and the impact of heterogeneity and publication bias was evaluated to be minimal throughout the analysis.

### 3.8 The effect of EMDR on anxiety symptom

As a result of analyzing the effect of EMDR on anxiety symptoms, a moderate and statistically significant treatment effect was observed in the group of patients with SUD (**Figure 3D**). Hedges’ g was estimated at 0.724, the standard error was 0.193, and the z value was 3.759, indicating a high level of statistical significance (p <.001). The results of the Omnibus test for all model coefficients were also statistically significant (Q(1) = 14.131, p <.001), suggesting that the effect of EMDR was consistent across studies. No significant heterogeneity was identified in the heterogeneity analysis. The residual heterogeneity test was not statistically significant (Q(3) = 2.100, p = .552), with I² at 0.0% and τ² estimated at 0.000, indicating a highly homogeneous pattern of results with minimal variation in effect sizes between studies.

Regarding potential publication bias, both Egger’s regression-based asymmetry test (z = 0.459, p = .646) and Kendall’s rank correlation test (τ = 0.000, p = 1.000) were not statistically significant, indicating a low likelihood of publication bias. Rosenthal’s fail-safe N was 17, suggesting that the results are unlikely to be overturned by unpublished studies with null findings. In summary, these findings demonstrate that EMDR produced a reliable and consistent treatment effect in reducing anxiety symptoms in patients with SUD. The overall pattern of results, along with the lack of substantial heterogeneity or publication bias, provides supportive evidence for the clinical utility of EMDR in treating anxiety as a primary target symptom.

## 4 Discussion

### 4.1 Summary of findings

In this meta-analysis, the effects of EMDR (Eye Movement Desensitization and Reprocessing) treatment on patients with SUD were evaluated across five primary symptom domains: craving, addiction severity, PTSD, depression, and anxiety. The results demonstrated moderate and statistically significant treatment effects for craving reduction (Hedges’ g = 0.55), PTSD symptom alleviation (g = 0.69), depression (g = 0.64), and anxiety (g = 0.72), with overall heterogeneity ranging from low to moderate across outcomes. In particular, heterogeneity was minimal in studies assessing anxiety and depression, and effect sizes in these domains were relatively consistent. In contrast, the effect of EMDR on addiction severity was not statistically significant (g = 0.14), and although moderate heterogeneity was observed, the influence of publication bias or influential outliers appeared negligible.

In the analysis of craving-related outcomes, participant characteristics (e.g., Alcohol/Drug Use Group vs. Nicotine Use Group) and study design type (randomized controlled trial vs. randomized crossover trial or quasi-experimental design) were identified as significant moderators of treatment effect sizes. According to the subgroup analysis presented in **Supplementary Figure S2**, the mean effect size observed in RCTs (Hedges’ g = 0.67, 95% CI: 0.46–0.88) was larger than that observed in randomized crossover and quasi-experimental studies (g = 0.38, 95% CI: 0.20–0.55). Furthermore, as shown in **Supplementary Figure S3**, the mean effect size in the SUD subgroup (g = 0.75, 95% CI: 0.50–1.00) was significantly larger than that in the smoking group (g = 0.38, 95% CI: 0.24–0.53).

These findings suggest that EMDR demonstrates a robust and consistent therapeutic effect on emotional comorbid symptoms (i.e., PTSD, depression, and anxiety) in individuals with SUD, and exhibits promising potential for ameliorating certain addiction-related outcomes, particularly craving. However, the present meta-analysis did not find a significant effect of EMDR on addiction severity. Given the limited number and methodological quality of the available studies, the evidence regarding this outcome remains inconclusive, highlighting the need for further rigorous clinical research in this domain.

### 4.2 Effects on addiction-related outcomes: craving and severity

We analyzed the effects of EMDR on craving and addiction severity, which are major addiction-related symptoms in patients with SUDs. The results of the analysis confirmed a moderate and statistically significant effect size (Hedges’ g = 0.55, 95% CI: 0.40–0.70) in reducing craving, and the results across studies were relatively consistent.

Most studies used an EMDR protocol that involves eliciting substance-related imagery or autobiographical memory, followed by eye movements with bilateral stimulation to reduce emotional responses and physiological arousal levels (35, 36, 44, 60). This approach represents an extension of the traditional TF EMDR, which targets emotionally maladaptive memories, and instead focuses on reducing the vividness and emotional intensity of craving-inducing images.

The theoretical basis for these effects can be explained through three major frameworks. First, the AIP model posits that symptoms arise from inadequately processed trauma- or addiction-related memories, and that EMDR facilitates their reprocessing (27). Second, according to the memory reconsolidation theory, reactivated memories enter a transiently labile state, during which they can be updated or modified with new information. EMDR may leverage this memory plasticity to desensitize and restructure addiction-related memories, thereby alleviating craving responses (37). Third, the working memory taxation theory suggests that performing bilateral stimulation (e.g., eye movements) while simultaneously holding vivid imagery in mind creates a competition for limited working memory resources. This interference reduces the vividness and emotional intensity of the imagery. Such a mechanism may also apply when reprocessing substance-related cues, helping to attenuate the intensity of cravings (36, 44).

Meanwhile, some studies have noted that EMDR’s effects on addiction-related outcomes are less consistent than those observed for PTSD (42). To address this, the present study conducted meta regression and subgroup analyses based on participant characteristics and study design. The results indicated that the mean effect size in RCTs (Hedges’ g = 0.67, 95% CI: 0.46–0.88) was significantly greater than that observed in randomized crossover or quasi-experimental studies (g = 0.38, 95% CI: 0.20–0.55). Additionally, the effect size in the SUD subgroup (g = 0.75, 95% CI: 0.50–1.00) was larger than in the smoking group (g = 0.38, 95% CI: 0.24–0.53). This discrepancy may be attributable to the fact that participants in the smoking group were not clinically diagnosed with nicotine dependence, but rather classified as daily or habitual smokers, and consequently exhibited lower baseline levels of craving than those observed in clinically diagnosed SUD populations (36, 37, 44). In fact, the study by Lemkes et al. (42) found no significant EMDR effect despite targeting inpatient populations. The researchers attributed this to the use of anti-craving medications in 26% of participants, and to the reduced exposure to craving-inducing stimuli in an inpatient setting (42). These findings suggest that baseline craving levels may play a critical role in moderating the effectiveness of EMDR. In addition, study design emerged as another influential moderator. The greater consistency of results observed in RCTs compared to non-randomized studies highlights the need for more rigorously controlled experimental designs in future research.

On the other hand, the effect of EMDR on addiction severity was not statistically significant in the present meta-analysis (Hedges’ g = 0.14, p = .42), a finding which carries important clinical implications. Similar findings have been reported in previous studies. For example, Sgualdini et al. (60) found no significant difference in relapse rates between the TAU and TAU+EMDR groups, attributing this to a limited sample size and a “floor effect” in which relapse rates in the control group were already low and thus could not be further reduced (60). Likewise, Callak Sarğın et al. (59) observed that initial reductions in craving completely dissipated within one month, underscoring the challenge of sustaining effects on more distal outcomes such as relapse or severity (59). These results suggest that addiction severity, as a long-term behavioral and clinical indicator, may be less responsive to short-term interventions compared to proximal outcomes such as craving or emotional distress (59, 60).

Moreover, addiction severity is closely intertwined with psychosocial vulnerabilities, including traumatic experiences, insecure attachment styles, and deficits in emotion regulation (9, 11, 12). These factors not only play a central role in the onset and maintenance of SUD but also make it more difficult to achieve lasting reductions in overall severity. For instance, meta-analytic evidence shows that individuals with SUD exhibit marked deficits in emotion regulation abilities, particularly in impulse control and strategy use (Hedges’ g =1.05) (61). Moreover, emotional dysregulation is robustly associated with more severe addictive behaviors and problem severity across substances (62). Theoretical and empirical work also links insecure attachment with impaired affect regulation, which in turn predisposes individuals to substance use as a maladaptive coping mechanism (12). Accordingly, the absence of significant improvements in addiction severity in this meta-analysis may indicate that treatment effects emerge first in domains more amenable to short-term change (e.g., craving, emotional reactivity) and may require more sustained or multidimensional interventions to be reflected in broader severity measures. Future research should therefore extend follow-up periods, recruit larger samples, and systematically examine how psychosocial factors such as trauma, attachment, and emotion regulation influence the trajectory of addiction severity.

These findings imply that although EMDR may be effective in temporarily modulating craving, its impact on long-term outcomes, intrusive imagery, and automatic thoughts remains limited. Thus, while AF EMDR may offer more direct effects on craving than conventional TF EMDR (59), systematic research incorporating longer-term follow-ups is warranted to determine the durability and generalizability of its effects.

It should also be noted that the included studies did not provide sufficient data to analyze outcomes of actual substance use, such as relapse rate or duration of abstinence. This represents an important limitation, as the ultimate clinical effectiveness of an addiction treatment should be evaluated by its ability to reduce substance use in real-world settings. The use of objective measures such as biomarkers or validated self-report instruments like the Timeline Follow-Back (TLFB) is crucial in this regard. Future research should therefore prioritize the systematic collection and reporting of these behavioral outcomes to provide a more comprehensive understanding of EMDR’s therapeutic effects on addiction.

### 4.3 Effects on comorbid PTSD symptoms in SUD

We evaluated the effect of EMDR treatment applied to patients with SUD on coexisting PTSD symptoms. As a result, the overall average effect size for PTSD-related symptoms was Hedges’ g = 0.69 (95% CI: 0.36–1.02), indicating a moderate and statistically significant treatment effect (**Figure 3B**).

SUD and PTSD exhibit a high rate of comorbidity in clinical populations. Approximately 30–60% of individuals with SUD seeking treatment meet diagnostic criteria for PTSD, and in some studies, over 90% report having experienced traumatic events (40, 41). This comorbidity extends beyond simple co-occurrence, leading to complex clinical outcomes, including chronic symptom trajectories, increased treatment dropout, elevated suicide risk, and heightened vulnerability to relapse due to the reciprocal interaction between the two disorders (63). For instance, hyperarousal and distress associated with PTSD may drive individuals to use substances in an attempt to self-medicate, while repeated substance use can interfere with PTSD recovery and intensify re-experiencing symptoms, thereby reinforcing a cycle of symptom maintenance and relapse vulnerability (63).

Within this framework, TF-EMDR primarily targets traumatic memories underlying PTSD, but symptom reduction in PTSD may indirectly alleviate substance-related problems. This mechanism helps to explain why TF-EMDR, although not specifically designed as an addiction-focused intervention, may still confer therapeutic benefits for SUD (40). A substantial proportion of individuals with SUD have experienced early-life trauma, such as emotional or physical abuse and neglect during childhood or adolescence, which may serve as key etiological factors in the development of addiction. Accordingly, TF EMDR interventions are increasingly recognized as a critical component of treatment for individuals with SUD, and effective resolution of PTSD symptoms may positively influence SUD outcomes (19, 40).

All studies included in this meta-analysis employed TF EMDR, which targets traumatic memories rather than addiction-related content. TF EMDR is designed to reprocess dysfunctionally stored traumatic memories, considered a core mechanism underlying PTSD symptoms. The treatment is based on the AIP model, which posits that symptomatology results from unprocessed distressing experiences. EMDR facilitates symptom reduction by reactivating traumatic memories within a safe therapeutic context and reprocessing them through bilateral stimulation, such as eye movements (27).

Moreover, several studies have reported that PTSD symptoms may begin to improve even prior to the completion of detoxification, indicating that early integration of EMDR into inpatient treatment may enhance overall therapeutic outcomes (19). Given the chronic and interactive nature of PTSD and SUD, these findings suggest that integrated, concurrent treatment approaches may be more effective than sequential or compartmentalized interventions (63).

Therefore, EMDR should not be regarded solely as a treatment for PTSD, but rather as a promising approach for addressing comorbid presentations of PTSD and SUD through an integrated and coordinated framework. However, since all PTSD related studies included in this meta-analysis employed TF-EMDR targeting traumatic memories rather than addiction-related content, the extent to which these findings can be generalized to AF-EMDR is limited. Future research should aim to systematically compare the effects of TF-EMDR and AF-EMDR on PTSD symptoms to clarify their distinct therapeutic contributions.

### 4.4 Effects on mood-related symptoms: depression and anxiety in SUD

This meta-analysis analyzed the effects of EMDR treatment on emotional symptoms such as depression and anxiety in patients with SUDs. As a result, the average effect size for depressive symptoms was Hedges’ g = 0.64 (95% CI: 0.25–1.03), and the average effect size for anxiety symptoms was Hedges’ g = 0.72 (95% CI: 0.35–1.10), indicating moderate and statistically significant treatment effects in both domains. In addition, there was little heterogeneity among the studies included in the analysis, suggesting that the effects of EMDR on improving emotional symptoms were relatively consistent across studies.

SUD patients commonly exhibit acute depressive and anxiety symptoms during the withdrawal and early recovery phases, which are associated with neuroendocrine dysregulation, including increased levels of adrenocorticotropic hormone (ACTH) and corticotropin-releasing factor (CRF) (40, 64). Due to this physiological vulnerability, individuals with SUD are more likely to experience complex and severe emotional disturbances compared to those with primary depressive or anxiety disorders. Recent animal study has investigated the neurobiological mechanisms associated with stress vulnerability (65). It has shown that EMDR may suppress hippocampal dendritic atrophy induced by excessive glucocorticoid exposure and can maintain hippocampal neuroplasticity (65). This finding suggests a potential neurobiological mechanism through which EMDR may alleviate depressive and anxiety symptoms by mitigating hippocampal dysfunction, a core contributor to emotional disturbances (65).

The beneficial effects of EMDR on such emotional symptoms have likewise been demonstrated in several human studies. For instance, research has shown that EMDR may modulate the heightened anxiety responses commonly observed during early withdrawal, thereby contributing to restoration of emotional stability (40). In particular, patients with higher scores on the Adverse Childhood Experiences (ACE) scale were found to exhibit greater reductions in anxiety and depressive symptoms, suggesting that the interaction between trauma history and EMDR-mediated processing may play a critical role in symptom alleviation (40, 66). Moreover, it has been proposed that negative emotions associated with addiction-related memories or mental imagery that elicit strong cravings, including self-reproach, hopelessness, and anxiety, may also be attenuated through the reprocessing mechanisms of EMDR (35). In other words, as emotionally overactivated addiction-related memories are reprocessed within a safe therapeutic context, the intensity of the emotional responses attached to these memories is concurrently reduced.

In addition, EMDR does not merely reduce the emotional distress of traumatic memories, but also facilitates the reconstruction of their meaning and the development of a more adaptive self-concept, thereby enhancing overall emotional regulation capacity (9). When the ability to recognize and regulate emotions is enhanced, the maladaptive emotional coping styles, such as impulsivity and avoidance, commonly observed in individuals with SUD may be mitigated, which may support more sustainable recovery outcomes. These findings suggest that EMDR is an effective integrative psychotherapy not only for posttraumatic stress symptoms but also for commonly co-occurring emotional symptoms (depression, anxiety, etc.) in SUD patients.

### 4.5 Limitations

This meta-analysis has several limitations. First, many of the included studies exhibited a high risk of bias, underscoring the need for more methodologically rigorous and reliable research. This is especially important given that the estimated effect size of EMDR varied depending on the type of experimental design. Second, detailed information on the treatment phase in which EMDR was delivered was generally lacking. As the timing of intervention (e.g., during detoxification, post-detox rehabilitation, or relapse prevention) may influence outcomes, the absence of such data prevented moderator analysis, highlighting an important direction for future research. Third, the number of included studies was limited across most outcome domains, preventing the implementation of meta-regression analysis except in the craving domain. This made it difficult to identify the sources of heterogeneity between studies or moderators influencing the effectiveness of EMDR. Fourth, the initial protocol of this study planned to examine clinical outcomes such as relapse rate or treatment adherence, but no study or only one reported these variables, making quantitative synthesis and reporting infeasible. In addition, most studies did not report standardized outcomes of actual substance use (e.g., abstinence duration or biomarkers), which limited our ability to evaluate the real-world clinical effectiveness of EMDR. Fifth, the sample size of most of the included studies was small, which may have overestimated the treatment effects, limiting the generalizability of the study results. Furthermore, we were not able to examine potential interaction effects between PTSD, mood, and substance use outcomes, as the included studies did not provide sufficient data to conduct such analyses. Nevertheless, given the well-established reciprocal relationships among these domains, whereby PTSD symptoms may exacerbate mood disturbances and substance use, and vice versa, future research should investigate these potential interactions to clarify the mechanisms through which EMDR exerts its therapeutic effects in SUD populations. Sixth, although a few studies reported long-term effects through follow-up observations, the majority evaluated only the immediate effects of EMDR. Furthermore, most of the studies included in this review did not differentiate between specific SUDs (e.g., alcohol, opioids, nicotine) or were limited in scope. Given that symptom profiles and treatment responses may differ across substance types (67, 68), future research should clearly distinguish between SUD subtypes and design clinical trials accordingly. In addition, studies focusing on populations with a high prevalence of SUDs but limited representation in current research, such as adolescents, women, correctional inmates, and minority groups, are warranted (69). Moreover, because only a few studies examined follow-up outcomes, a meta-analysis could not be performed to evaluate the sustainability of long-term treatment effects. These limitations underscore the need for future research that employs more rigorous methodologies, incorporates both immediate and sustained outcome assessments, and systematically addresses variability across SUD subtypes and populations. Such efforts will contribute to a more precise understanding of the therapeutic effectiveness of EMDR and broaden its potential for practical clinical application.

## 5 Conclusion

This meta-analysis comprehensively evaluated the therapeutic effects of EMDR therapy on patients with SUD. The results indicated that EMDR yielded significant therapeutic effects of moderate or greater magnitude on craving, PTSD, depression, and anxiety symptoms, with overall heterogeneity ranging from low to moderate. In particular, the effect on craving varied according to the clinical characteristics of the subjects (SUD vs. smokers) and the type of study design (RCT vs. non-randomized study), suggesting that EMDR may be clinically applicable not only to emotional comorbid symptoms but also to certain addiction-related symptoms. By contrast, the effect on addiction severity was not statistically significant, and the evidence regarding long-term outcomes remains limited.

This study sought to overcome the limitations of prior meta-analyses by adopting a more rigorous analytic approach, including effect size estimation based on pre-post change scores and moderator analyses. Through this, it offered practical implications for the potential clinical application of EMDR and the development of tailored intervention strategies based on patient characteristics. However, the generally low methodological quality of the included studies and the limited number of studies available for analysis were shortcomings of this study. In particular, the evidence for long-term treatment effects and key clinical outcomes was insufficient, limiting conclusions regarding the sustained efficacy of EMDR.

Therefore, future studies should adopt more rigorous research designs with adequate sample sizes and extended follow-up periods. In addition, more nuanced study designs that consider potential moderating variables, such as baseline craving levels, type of EMDR intervention (AF vs. TF), and comorbidity patterns, are warranted. Such research will help to further clarify the therapeutic potential of EMDR and inform its practical application in the treatment of substance use disorders.

## Data Availability

The original contributions presented in the study are included in the article/**Supplementary Material**. Further inquiries can be directed to the corresponding author.
