# Supplementary material for: Therapeutic effects of eye movement desensitization and reprocessing for substance use disorders: a meta-analysis of addiction-related and emotional symptoms
Source: Front Psychiatry. 2025 Sep 17;16:1660046. doi: 10.3389/fpsyt.2025.1660046 (PMC12484161; doi:10.3389/fpsyt.2025.1660046)
Supplement: Supplementary file 1 [file DataSheet1.docx]

**Supplementary Materials**


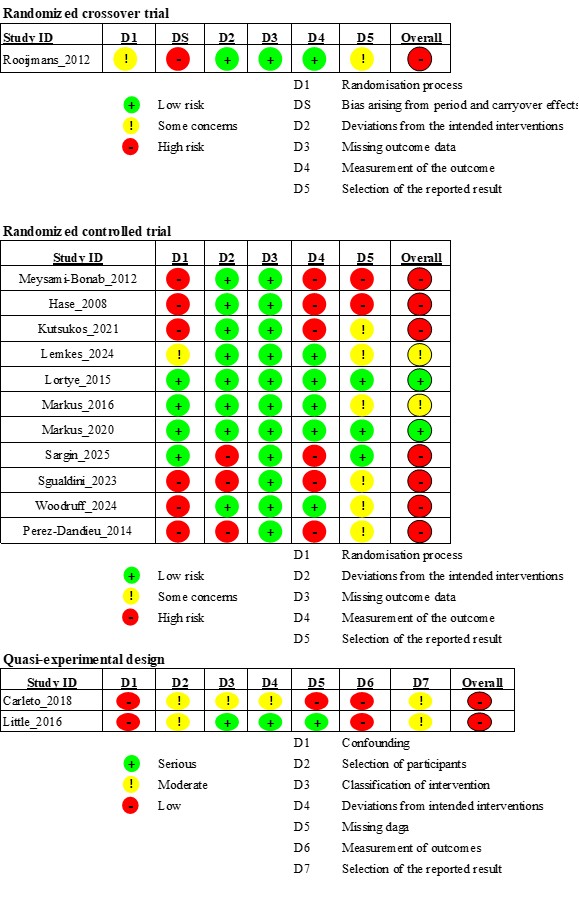


Figure S1. Risk of Bias Assessment for Included Studies


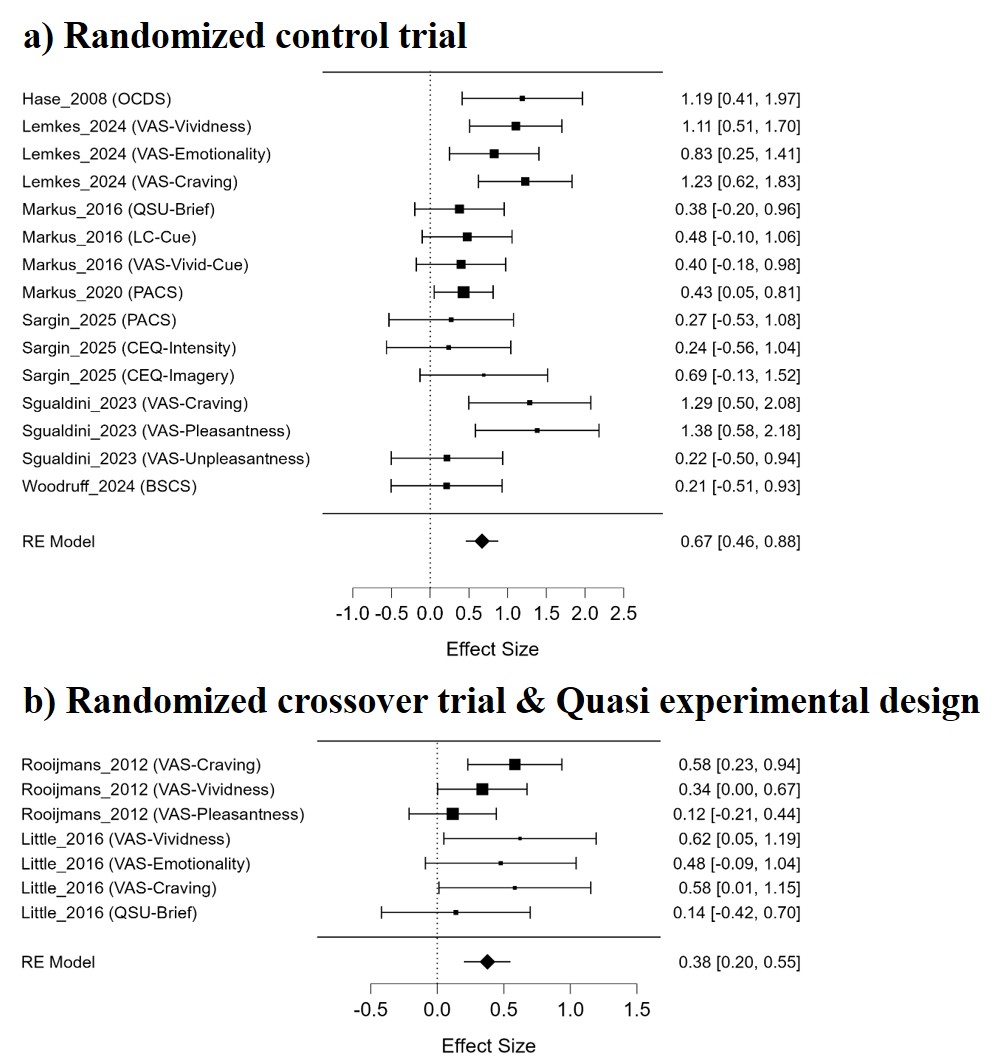


Figure S2. Subgroup Analysis by Study Design


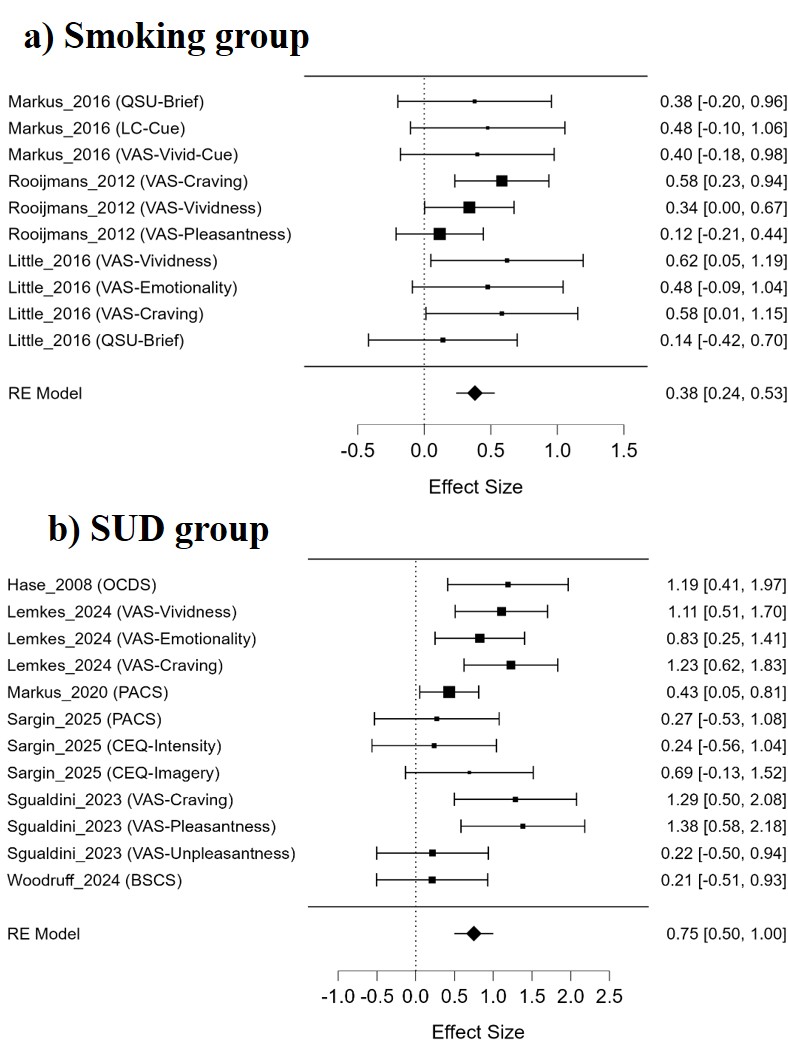


Figure S3. Subgroup Analysis by Participant Group
